# Economic support to improve tuberculosis treatment outcomes in South Africa: a pragmatic cluster-randomized controlled trial

**DOI:** 10.1186/1745-6215-14-154

**Published:** 2013-05-28

**Authors:** Elizabeth Lutge, Simon Lewin, Jimmy Volmink, Irwin Friedman, Carl Lombard

**Affiliations:** 1 Faculty of Health Sciences, Stellenbosch University, Francie van Zijl Drive, Tygerberg, 7505, South Africa; 2 Health Systems Research Unit, Medical Research Council of South Africa, Francie van Zyl Drive, Parrow, 7505, South Africa; 3 Norwegian Knowledge Centre for the Health Services, PO Box 7004 St. Olavs plass, Oslo, N-0130, Norway; 4 Cochrane Centre, Medical Research Council of South Africa, Francie van Zyl Drive, Parrow, 7505, South Africa; 5 Health Systems Trust, 34 Essex Terrace, Westville, 3630, South Africa; 6 Biostatistics Unit, Medical Research Council of South Africa, Francie van Zyl Drive, Parrow, 7505, South Africa

**Keywords:** Conditional cash transfers, Economic support, Incentives, Poverty, TB

## Abstract

**Background:**

Poverty undermines adherence to tuberculosis treatment. Economic support may both encourage and enable patients to complete treatment. In South Africa, which carries a high burden of tuberculosis, such support may improve the currently poor outcomes of patients on tuberculosis treatment. The aim of this study was to test the feasibility and effectiveness of delivering economic support to patients with pulmonary tuberculosis in a high-burden province of South Africa.

**Methods:**

This was a pragmatic, unblinded, two-arm cluster-randomized controlled trial, where 20 public sector clinics acted as clusters. Patients with pulmonary tuberculosis in intervention clinics (n = 2,107) were offered a monthly voucher of ZAR120.00 (approximately US$15) until the completion of their treatment. Vouchers were redeemed at local shops for foodstuffs. Patients in control clinics (n = 1,984) received usual tuberculosis care.

**Results:**

Intention to treat analysis showed a small but non-significant improvement in treatment success rates in intervention clinics (intervention 76.2%; control 70.7%; risk difference 5.6% (95% confidence interval: -1.2%, 12.3%), *P* = 0.107). Low fidelity to the intervention meant that 36.2% of eligible patients did not receive a voucher at all, 32.3% received a voucher for between one and three months and 31.5% received a voucher for four to eight months of treatment. There was a strong dose–response relationship between frequency of receipt of the voucher and treatment success (*P* <0.001).

**Conclusions:**

Our pragmatic trial has shown that, in the real world setting of public sector clinics in South Africa, economic support to patients with tuberculosis does not significantly improve outcomes on treatment. However, the low fidelity to the delivery of our voucher meant that a third of eligible patients did not receive it. Among patients in intervention clinics who received the voucher at least once, treatment success rates were significantly improved. Further operational research is needed to explore how best to ensure the consistent and appropriate delivery of such support to those eligible to receive it.

**Trial registration:**

Current Controlled Trials ISRCTN50689131

## Background

There is a large body of work, dating back over a century, that emphasizes the close relationship between poverty and tuberculosis (TB). Indeed, Robert Koch himself described the disease as ‘the outcome of social misery’ [1]. Rene and Jean Dubois, who authored the seminal book ‘The White Plague: Tuberculosis, Man and Society’ called TB ‘a social disease’ [2]. The prevalence of TB is higher in poorer countries [3] and among poorer communities in wealthy countries [4]. Indeed, in South Africa, a country of profound income inequality [5], TB has been called a ‘barometer of poverty’ [6].

South Africa has one of the highest burdens of TB in the world, which can be attributed, at least in part, to conditions of poverty that favor its transmission [7]. In addition, the high prevalence of HIV infection has played a central role in increasing and maintaining the high burden of TB in the country and may also be responsible for undermining outcomes of patients on TB treatment. The association between poverty and poor adherence to anti-retroviral treatment [8,9] has profound implications for the role of HIV in adherence to anti-TB treatment and for outcomes of patients on that treatment. At present, the outcomes of patients on TB treatment in South Africa remain below the targets set by the World Health Organization [10]. Although these outcomes have improved over the last decade, the transmission rate of TB has increased dramatically over this time and it has been suggested that the current national strategy for TB control, based on the Directly Observed Treatment Strategy, is insufficient to control the epidemic [11].

The association between poverty and TB exists over the entire course of the disease [12]. Although effective treatment is available for drug-susceptible TB, and this is provided free of charge in public sector clinics in South Africa, there is a wealth of research that shows that the effect of poverty on TB outcomes is due, at least in part, to the costs of accessing and adhering to treatment [13-17]. In addition, the poor nutrition that often accompanies poverty is not only a risk factor for the development of TB [18-20], but undermines the outcomes of those on TB treatment [21].

Although the association between poverty and TB is well documented, there are very few programs which directly address this relationship with economic interventions, and even fewer research studies which evaluate them [12]. Such research is difficult to do. Because of the scale on which poverty occurs, its inherent complexity and the complex relationship between different aspects of poverty and disease, such studies may be difficult to design and enormously costly to conduct. Addressing this point, the Commission on the Socio-economic Determinants of Health recently noted that, in spite of the growing body of evidence to support action in this field, ‘there is a pressing need to invest in a great deal more research, bringing together different disciplines and areas of expertise, to work out how social determinants create health inequity, and how action on these determinants can produce better, fairer health’” [22].

The call for research in the field of economic support for improving TB outcomes has been echoed by several authors who have conducted reviews on the effects on health of results-based financing [23], conditional cash transfers [24], economic incentives [25], and cash transfers and microfinance [26]. A recent expert consultation on Social Protection Interventions for Tuberculosis Control, held at Chatham House in London in February 2012, concluded that ‘a) despite the indirect evidence gathered in a recent review from Boccia *et al*. (2011) [26], the actual impact of social protection on TB indicators (e.g. incidence, mortality, case finding, TB treatment adherence) remains unknown; b) it is unclear how social protection initiatives may be best integrated with current TB control activities and which forms of social protection are most likely to be successful, depending on the objectives posed’ [27].

Our trial aimed to generate evidence on both of these areas. It aimed to investigate the feasibility of delivering a form of economic support to patients with TB as an integrated part of the TB control program of government-run clinics in South Africa, and to determine whether such support was effective in improving the outcomes of patients with TB in these clinics.

## Methods

### Study setting

This study was conducted in KwaZulu-Natal, one of South Africa’s poorest and most populous provinces. The province has the second highest population and the second highest population density of the country, including the highest number of children under the age of one year [10]. The poverty rate amongst individuals in KwaZulu-Natal is the second highest in the country, at 58.5%, and just over a quarter (25.5%) of all poor individuals in the country live in the province [28].

KwaZulu-Natal has the highest incidence of TB in South Africa (1,142 per 100,000) as well as the highest HIV prevalence rate nationally [10]. Probably because of its high burden of HIV, the province has for several years held the dubious honor of having the highest number of patients with TB in the country (120,421 cases of all types of TB in 2010) [10]. TB is the most important cause of death in the province, causing 16.2% of all deaths in 2009 [29].

At the request of a senior TB program manager during the planning phase of the trial, our trial included one urban district and one rural district.

### Study design

This was a pragmatic, unblinded two-arm cluster-randomized controlled trial, using primary health care clinics as clusters. Nested within the controlled trial was a process evaluation, consisting of in-depth interviews with research participants, and an assessment of the effect of the voucher on patient household expenditures (to be reported elsewhere).

A cluster-randomized design was chosen for this trial because we felt it would be logistically easier for nurses to deliver the intervention to all of their TB patients (as opposed to a selected number) [30], and because we hoped to avoid creating resentment among patients who did not receive the intervention. In addition, to test whether the administration of the intervention was feasible as a routine part of the TB control program in South Africa, this trial had a pragmatic nature [31]. Distinct from an explanatory trial, which measures efficacy (‘the benefit a treatment produces under ideal conditions’), a pragmatic trial measures effectiveness (‘the benefit the treatment produces in routine clinical practice’ [32]). Because trials are seldom purely pragmatic or explanatory [33], their position on the continuum between these can be described by the relative orientation of the building blocks of the trial. The ‘dimensions’ or ‘domains’ of the pragmatic-explanatory continuum indicator summary (PRECIS) tool are useful measures that give a holistic picture of the general pragmatic or explanatory nature of the trial [34]. The orientation of the domains of this trial are presented in Additional file 1.

### Study setting and participants

#### Clinics

Public sector (government-funded) primary care clinics were chosen for this study because these treat the majority of patients with TB in South Africa. These clinics are managed by professional nurses, and staffed by nurses of all grades.

**Figure 1:**
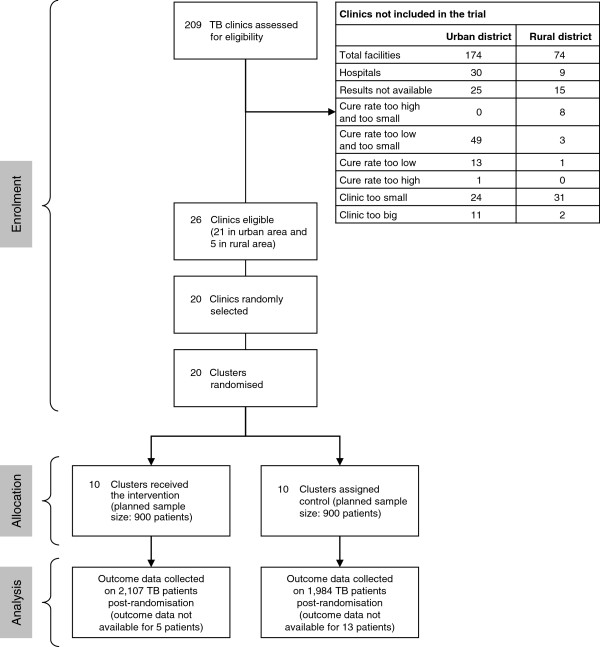
Participant flow diagram.

Clinics with cure rates of between 40% and 70% for the year preceding the trial were eligible for inclusion in the study. The upper limit was set because demonstrating a clinically meaningful effect in clinics with cure rates higher than 70% would have required a very large sample size. The lower limit was set to reduce between-clinic variability and to exclude clinics where poor service provision and systemic weaknesses may have contributed to poor cure rates. Nine clinics were excluded because their cure rates were too high, and 66 were excluded because their cure rates were too low. In addition, only clinics seeing between 20 and 150 new patients with smear-positive TB per year were eligible for inclusion. The lower limit was set to meet the sample size requirements of the trial, and the upper limit was set because implementing the intervention in very large clinics would have exceeded the limited budget of this trial. Fifty-five clinics were excluded because they were too small, and 13 clinics because they were too big. These criteria meant that, of a total of 209 public sector clinics providing TB care in the districts chosen, only 26 (12%) were eligible for inclusion in this trial (Figure 1).

#### Patients

All patients diagnosed with pulmonary, drug-sensitive TB and attending intervention clinics within the period 1 July 2009 to 31 March 2010 were recruited into the trial; however, only patients who started TB treatment within this recruitment period were eligible for analysis. Patients were followed up to the end of their treatment (which is a maximum of six months in new cases and eight months in re-treatment cases). Both adults and children were included in the analysis because, as a high-burden country, there are a significant number of children receiving treatment for TB in South Africa.

#### Intervention

A voucher, valued at ZAR120 (approximately US$15) was offered to patients by nurses every month on collection of their treatment, to a maximum of eight months. A voucher was preferred to cash by TB program managers and other stakeholders with whom the study was discussed, because it would be a security risk to hold large sums of cash at clinics in KwaZulu-Natal; most patients at public sector clinics would not have bank accounts, thus making electronic transfers difficult; cash may be spent on any items, and patients may have chosen to spend it on unhealthy or damaging items such as cigarettes or alcohol; and the expenditure of vouchers could be more easily monitored.

The value of ZAR120 was chosen because it was considered by TB program managers to be too small to act as a perverse incentive for patients to remain ill, but large enough to encourage patients to adhere to treatment. The value of the voucher was lower than the food poverty line of ZAR226 at the time of the study [35], and was about a fifth of the value of the median per capita income in KwaZulu-Natal around the time of the trial [36]. However, the amount was sufficient to purchase a number of food stuffs commonly used in South African households (Table 1). It was therefore hoped that the voucher would allow households to increase expenditure on food stuffs and so improve household food security and the nutritional status of the index patient. It was also hoped that the voucher would free up money spent on food stuffs to meet other essential expenditure, such as transport to the clinic.

**Table 1:** **Prices of selected food stuffs commonly used in South African households, January 2010 (mid-way through trial)**[37]

**Commodity**	**Rural price in ZAR**	**Urban price in ZAR**
Full cream long life milk (1 L)	10.28	9.72
Loaf of brown bread (700 g)	7.00	6.97
Loaf of white bread (700 g)	7.56	7.83
Maize meal (5 kg)	29.09	22.93
Margarine (500 g)	14.61	12.88
Peanut butter (400 g)	16.59	15.48
Rice (2 kg)	28.58	23.14
Sunflower oil (750 mL)	17.20	12.81
Ceylon/black tea (62.5 g)	7.06	7.02
White sugar (2.5 kg)	19.73	18.15

The vouchers were redeemable at specific general stores, chosen by the nurses at each participating clinic on the basis of their proximity to the clinic and the relative costs of their goods. At the request of TB program managers, patients were advised by clinic nurses to spend the vouchers on healthy foodstuffs; however, nurses did not monitor their expenditure and the stores were asked to allow patients to purchase any goods up to the value of the voucher.

Patients at control clinics received usual TB care.

#### Logistics of voucher delivery

Nurses at intervention clinics were supplied with voucher books. Each voucher had a unique number and was carbonized so that the information entered by the nurse onto the top copy would appear on two other copies below. The nurse entered the patient’s name, gender, identity number and clinic number onto the voucher, signed it and stamped the top copy with the clinic stamp. A unique sticker was placed on each voucher, without which it could not be redeemed, to minimize the risk of fraudulent copies of vouchers being made and used. The nurse gave the patient the top two voucher copies and kept the final copy at the clinic. The patient took the two copies of the voucher to the designated shop and exchanged these for goods. Both copies of the voucher were retained by the shop. Every four to six weeks, the principal investigator collected one copy of the vouchers from the shop, and used these to calculate the amount owed to the shop for goods purchased. The shops kept one voucher copy for their own records. The clinic copies were also collected to ensure that the vouchers distributed tallied with the vouchers redeemed.

Patients were asked to present their identity books or clinic cards on redemption of the vouchers. The presentation of an identity book was not mandatory as some patients did not possess this document. Relatives or friends were permitted to redeem vouchers on behalf of very sick patients provided they presented the patients’ clinic cards along with the vouchers. Vouchers had to be redeemed within one month of acquisition, and could not be exchanged for cash. No change was given by the shops if the full amount was not spent, although patients could supplement the vouchers with their own cash. The till slips, indicating the purchases made and their prices, were attached by cashiers to the redeemed vouchers and collected with the vouchers by the principal investigator. The data on patient purchases was analyzed and will be reported elsewhere.

#### Support from study team for voucher delivery

At the outset of the trial, investigators showed the TB nurses at each intervention clinic how to administer the voucher. Each nurse was given a study information sheet containing this information. If nurses were replaced, outgoing nurses explained the process to new nurses and this was reinforced by an additional visit from the principal investigator. Nurses were able to telephone the principal investigator at any time to discuss queries about the voucher or its delivery. The investigators visited each intervention clinic every four to six weeks to collect the vouchers from the TB nurses and to discuss any problems that might have arisen in the trial.

Similarly, managers of participating shops were shown how the voucher was to be used at the outset of the study. Managers were responsible for training cashiers in the use of the voucher. Shops were visited by the investigators every four to six weeks and at these visits, any problems with the administration of the voucher were discussed.

### Ethics and consent

Ethical approval for the trial and its process evaluation was received from the Committee for Human Research at the University of Stellenbosch (N07/10/245). Permission to conduct the trial was received from the KwaZulu-Natal Provincial Department of Health, the participating District Offices, the Research Committee of the eThekwini Municipality and the sisters in charge at individual clinics. In addition, as ‘guardians’ of the communities in which the trial took place, permission to conduct the trial was received from the Health and Safety Sub-Committee of elected City Councilors in the urban district and from individual ward councilors and traditional leaders of the isigodi (wards) where rural clinics were situated. Although all participants in the main trial were informed verbally and in writing of the study, individual written consent from patients in intervention clinics was not sought because the research was considered to be very low risk, and because obtaining informed consent from each individual might have made the project unfeasible [30]. Waiver of written informed consent from patients in intervention clinics was approved by the Committee for Human Research at the University of Stellenbosch before the trial started. Individual written informed consent was obtained for all participants in the process evaluation.

### Registration

This trial is registered with Current Controlled Trials (reference ISRCTN50689131), the South African Clinical Trials Registry (reference DOH-27-0409-2791), the Wellcome Trust Register of Clinical Trials (reference 083619) and the Pan African Clinical Trials Registry (reference PACTR2010010001275437).

The trial protocol is available at the following web address: http://www.hst.org.za/publications/study-protocol-economic-incentives-improving-clinical-outcomes-patients-tb-south-africa.

### Outcomes

The primary outcome was TB treatment success, defined as the sum of those patients cured and those completing treatment. Secondary outcomes were default and treatment failure rates. Data on other routine TB treatment outcomes were also collected, although they were not in the original protocol. The World Health Organization definitions for these outcomes, which are used in South African clinics and were used for the purposes of this study, are listed in Additional file 2.

Outcomes were ascertained by participating clinics using their usual procedures. In smear-positive patients, this was done through sputum microscopy and culture after six months of treatment in new patients and after eight months of treatment in re-treatment patients. In smear-negative patients, where initial diagnosis is based on chest X-rays and clinical signs, cure cannot be determined and treatment completion is the outcome used [38]. No additional clinical investigations were performed for the purposes of this study.

Data on factors that have been shown to impact on adherence to treatment [14,39] were also collected. These factors were age, gender, employment status, type of TB and HIV status (determined by PCR or ELISA tests).

Clinic TB registers and individual patient files (held at the clinics) and electronic TB registers (held at participating district offices) were used as sources for all these data. Patient files are completed by the consulting nurse, and clinic registers are completed in many cases by a clerk attached to the TB program in the clinic. The latter are checked by the nurse or sister in charge of TB at the clinic. In a few small clinics, where clerks are not available, this record keeping is done by the nurse working in TB. In this study, the clinic register was the primary source of data. Patient files were used to fill in missing data for any patients whose information in the clinic or electronic registers was incomplete. Data obtained from clinic registers were checked for accuracy by comparing the outcomes obtained with those in a sample of individual patient files. A 10% random sample of data from the total trial population was checked. This was done by randomly identifying a starting point on the trial database, and retrieving every tenth patient file for those patients from the clinic.

### Statistical methods

#### Sample size

At the time of the study, there were a total of 144 clinics in the urban and 65 clinics in the rural district providing TB care. A list of those clinics meeting the inclusion criteria was constituted. Twenty-one clinics in the urban and five in the rural district were eligible for inclusion.

An intra-cluster correlation co-efficient of 0.03 was calculated based on pre-trial data from the clinics. To detect a 15% difference in treatment success rates (which we felt would be the minimum difference required to influence policy), based on a power of 90%, at a significance level of 5% (two-sided test) and an average cluster size of 100, 18 clinics were necessary. Twenty clinics were included in the sample to allow for clinic drop out during the trial.

#### Randomization

The 20 study clinics were randomly selected from the 26 eligible clinics stratified by district. Sixteen study clinics were selected in the urban and four in the rural district. Within the two districts, the study clinics were randomly assigned in a 1:1 ratio, using a randomization list generated by the study statistician.

Clinics were allocated to intervention or control groups by the study statistician and no changes were made to this allocation. Staff members in all clinics were aware that they were part of a trial to test the effectiveness of a voucher.

Clinics were enrolled by the principal investigator, and participants within the clinics were enrolled by the nurses in charge of TB care at each clinic.

#### Blinding

Because of the nature of the intervention, no blinding was possible in this study. Data extractors were not blinded as it was considered neither practical nor feasible to conceal from them the intervention status of the clinics from which they collected data.

#### Analysis

Analysis was by intention to treat and patient level data was used for this purpose. For the binary study outcome (treatment success achieved, not achieved) a generalized linear model (GLM) was used to evaluate the intervention effect with adjustment for the stratification of clinics at randomization. The clustering effect of clinics was taken into account through cluster robust variance estimation.

An exploratory analysis was also performed for the primary outcome. In this analysis, the intervention group was limited to participants who received at least one voucher. The control group remained unchanged.

As a secondary analysis of the intention to treat study population, a multiple regression model (GLM) was done to investigate the impact of adjusting for selected covariates on the estimated intervention effect. The covariates included in this model were employment status, whether the participant was a minor, whether the TB type was smear positive or diagnosed clinically or on X-ray, and gender. The model was evaluated for interaction effects between the intervention and any of the covariates.

A further secondary analysis was done to test for a dose–response effect in the intervention arm. The study outcome was evaluated against the number of months the participant had received a voucher. The GLM approach was used for this purpose [40].

## Results

### Participant flow

As seen in Figure 1, there was no loss of clinics in the trial, and all eligible patients in each clinic were included in the analysis. Loss to follow-up was small, with outcome data unavailable on 0.2% of patients in intervention clinics and 0.7% of patients in control clinics.

### Recruitment

Recruitment of patients took place over eight months (July 2009 to March 2010 inclusive). Patients who started treatment during this period were followed up to the end of their treatment. The trial ended on 30 September 2010, when the last recruited patients completed their full course of treatment.

### Baseline data

A total of 4,091 patients were included in this study: 1,984 in the control arm and 2,107 in the intervention arm. The number of patients enrolled by clinics varied between 68 and 335.

The baseline characteristics of the two groups were calculated and are presented in a comparative table (Table 2).

**Table 2:** Baseline characteristics of trial cohorts

	**Intervention clinics**	**Control clinics**
Total number of trial participants	2,107	1,984
Minimum number of participants per clinic	122	68
Maximum number of participants per clinic	335	335
Mean age of participants	29 years	32 years
Number (percentage) of male participants	1,058 (50.2%)	1,069 (53.9%)
Number (percentage) of participants in rural district	266 (12.6)	167 (8.4)
Number (percentage) of HIV-positive participants	910 (68.0%)	1106 (73.0%)
Number of unemployed participants	1 081 (60.2%)	1 228 (66.2%)
Number of child participants (less than 13 years)	386 (21.5%)	251 (13.5%)
Number of smear-positive participants	903 (42.9%)	882 (44.5%)

Data on HIV status and employment were not available for all patients.

### Primary outcomes

Intention to treat analysis showed a small and non-significant improvement in treatment success rates in the group receiving the vouchers. The exploratory analysis showed a larger and significant improvement in treatment success rates in the intervention arm (Table 3).

**Table 3:** Primary outcome (treatment success) - intention to treat and exploratory analyses

**Outcome**	**Intervention group (%)**	**Control group (%)**	**Risk difference (%) (95% confidence interval)**^**a**^	***P***
Treatment success/Intention to treat analysis	1,606/2,107 (76.2)	1,402/1,984 (70.7)	5.6 (−1.2, 12.3)	0.107
Treatment success/Exploratory analysis	1,051/1,294 (81.2)	1,402/1,984 (70.7)	10.6 (3.7, 17.5)	0.003

The estimated intra-cluster correlation co-efficient for our study for the primary outcome was 0.033. This is very close to the intra-cluster correlation co-efficient assumed in the sample size calculation (0.03). ^a^Estimate from generalized linear model.

There was greater variability in the outcomes of clinics in the control arm compared with the intervention arm. There were four clinics in the intervention arm with treatment success rates of more than 80%, compared with only one in the control arm, and three clinics in the control arm with treatment success rates of less than 65% compared with none in the intervention arm (Table 4).

**Table 4:** Treatment success per clinic (intention to treat analysis)

**Intervention clinics**			**Control clinics**		
**Clinic**	**Treatment success (%)**	**Other TB outcome (%)**	**Clinic**	**Treatment success (%)**	**Other TB outcome (%)**
1	202 (80.16)	50 (19.84)	1	88 (89.80)	10 (10.20)
2	238 (78.55)	65 (21.45)	2	99 (52.66)	89 (47.34)
3	198 (68.99)	89 (31.01)	3	112 (58.03)	81 (41.97)
4	198 (68.99)	89 (31.01)	4	240 (71.64)	95 (28.36)
5	83 (68.03)	39 (31.97)	5	204 (73.81)	72 (26.09)
6	274 (81.79)	61 (18.21)	6	159 (68.24)	74 (31.76)
7	82 (66.67)	41 (33.33)	7	140 (72.16)	54 (27.84)
8	183 (81.33)	42 (18.67)	8	233 (77.67)	67 (22.33)
9	106 (83.46)	21 (16.54)	9	85 (85.86)	14 (14.14)
10	104 (74.82)	35 (25.18)	10	41 (60.29)	27 (39.71)

### Secondary outcomes

The treatment completion rates of patients in the intervention arm were almost 10% higher than those in the control arm; however, the cure rates of patients in the intervention arm were slightly lower than those in the control. Default, treatment interruption and treatment failure rates were all lower in the intervention arm (Table 5).

**Table 5:** Tuberculosis treatment outcomes for patients in intervention and control clinics

**Treatment outcome**	**Intervention group (%)**	**Control group (%)**
	**(n = 2,107)**	**(n = 1,984)**
Treatment completed	911 (43.2)	694 (35.0)
Cured	695 (33.0)	708 (35.7)
Defaulted	158 (7.5)	202 (10.2)
Treatment interrupted	0 (0.0)	15 (0.8)
Treatment failure	79 (3.8)	113 (5.7)
Multi-drug resistant TB	1 (0.1)	3 (0.2)
Died	151 (7.2)	137 (7.0)
Moved/transferred	107 (5.1)	99 (5.0)
No outcome data available	5 (0.2)	13 (0.7)

### Ancillary analysis

The adjusted analysis (Table 6) reflects the intervention effect in the presence of possible confounders (excluding HIV status because the missing data for this variable was too extensive). The intervention effect shown (4.6%) is slightly smaller than in the unadjusted intention to treat analysis (5.5%). Thus about 1% of the intervention effect in the unadjusted analysis can be explained by differences in the participants of the intervention and control clinics.

**Table 6:** Regression model showing patient characteristics associated with treatment success (generalized linear model)

**Patient characteristic**	**Number/Total for whom data for this variable was available**	**Adjusted risk difference**	**95% confidence interval**	***P***
Intervention group indicator	2107/4091	0.046	−0.017, 0.109	0.153
Unemployed (yes)	2309/3650	−0.044	−0.092, 0.005	0.077
Child (yes)	637/3650	0.075	0.026, 0.124	0.003
Smear-positive TB indicator	1785/3614	0.04	0.008, 0.072	0.014
Female	1949/4076	0.032	0.008, 0.056	0.014
Intercept^a^		0.718	0.643, 0.725	<0.001

^a^The intercept represents the outcome in the control arm when all other covariates = 0.

Patients who were unemployed had significantly lower treatment success rates than those who were employed. Children under the age of 13 had significantly better treatment success rates than those over 13 years, women had better rates than men and patients with smear-positive TB had significantly better treatment success rates than those with smear-negative TB. No significant interactions between any of these subgroups were found (Table 6).

### Adherence to the intervention

Of all 2,076 patients who were eligible to receive a voucher for the six to eight months of their treatment, 813 (36.2%) did not receive a voucher at all, and 671 (32.3%) received a voucher for between one and three months. The remainder received a voucher for four to eight months of treatment. In many cases, nurses in intervention clinics withheld vouchers from eligible patients whom they felt were relatively better off financially (process evaluation, to be reported elsewhere). This preference for giving vouchers to patients who were relatively more deprived is illustrated in an analysis of eligible patients who received at least one voucher compared to eligible patients who did not receive any vouchers at all. In this group, there were significantly more unemployed patients who received vouchers (*P* = 0.04), whilst there were significantly fewer children who received vouchers (*P* = 0.03) (Table 7). In addition, women were more likely to receive vouchers than men (*P* = 0.026).

**Table 7:** Comparison of eligible patients who received vouchers with eligible patients who did not

**Received at least one voucher**	**Employed (%)**	**Children (%)**	**Pensioner (%)**	**Student (%)**	**Unemployed (%)**	**Total (%)**
No	117 (17.06)	178	12	2	377	686
		(25.95)	(1.75)	(0.29)	(54.96)	(100.00)
Yes	172	202	16	6	691	1,087
	(15.82)	(18.58)	(1.47)	0.55	(63.57)	(100.00)

**Figure 2:**
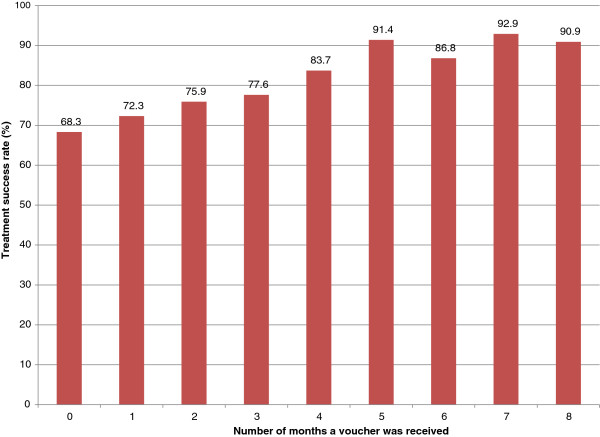
Effect of increasing frequency of vouchers on treatment success rate.

The effect of the vouchers was to increase treatment success rates in unemployed patients from 67% in the control arm to 71.7% in the intervention arm. In the remaining patients the treatment success rate was 76.7% in the control arm versus 82.8% in the intervention arm. It is clear that there was no interaction effect; the intervention boosted treatment completion rates in both groups equally. Further analysis of unemployed patients in the intervention group showed that eligible unemployed patients who did not receive a voucher achieved a 60% treatment success rate compared with 77.6% of the eligible unemployed patients who did receive a voucher. There was a strong dose–response effect (*P* <0.001) (Figure 2). The treatment success rate of patients who did not receive any vouchers was 68.3%, compared with a rate of more than 90% in patients who received a voucher for five months or more.

### Adverse events

The adverse events investigated in this study were those related to the voucher, and not to the clinical consequences of TB or its treatment. Specifically, there were very few reports of patients spending the vouchers on alcohol or cigarettes (assessment of expenditure of vouchers, to be reported elsewhere), ‘leakage’ of vouchers, or coercion of staff by patients to give them a voucher (process evaluation, to be reported elsewhere). However, some staff were concerned that the vouchers would create dependency and incentivize non-adherence (process evaluation, to be reported elsewhere). Also, some patients reported in interviews that when relatives or friends had redeemed vouchers on their behalf, the relatives had not given them (the patients) the goods. Finally, those patients who were not eligible to receive the vouchers in intervention clinics (that is, those with extrapulmonary TB) expressed varying degrees of anger about this, both to clinic nurses and to the principal investigator (process evaluation, to be reported elsewhere).

## Discussion

This was the first trial in Africa to investigate the effect of economic support (a monthly voucher) on the outcomes of patients on TB treatment. The trial found a 5.6% improvement in treatment success rates among patients who received the voucher, meaning that for every 1,000 patients who received the voucher, an additional 56 would have achieved treatment success. This was lower than the 15% difference that the study was powered to detect, which explains in part, the failure of the trial to achieve a significant result. This failure may be further explained by low fidelity to the intervention, which is discussed further in the process evaluation (to be reported elsewhere). The exploratory analysis, which compared patients in intervention clinics who had received at least one voucher to the control group, showed significantly higher treatment success rates in intervention compared to control clinics. A powerful dose–response effect was demonstrated, with patients who received vouchers more frequently being more likely to complete treatment.

This trial aimed both to reward adherence behavior, and to make adherence easier by ameliorating two features of poverty which are commonly associated with TB: under-nutrition and limited access to health care [12]. We hypothesized that the voucher (if used for purchasing food) would improve patients’ food security and release household funds for use elsewhere, such as for transport to the clinic [14]. In 2008, 71% of the households in KwaZulu-Natal lived on less than 40% of the median per capita income of ZAR569.00 per month [36]. This suggests that, although the value of the voucher was small relative to the median per capita income at the time of the trial, the voucher may nonetheless have facilitated a substantial improvement in the food purchases of households.

The evidence for the efficacy or effectiveness of economic support in improving the outcomes of patients on TB treatment is slim. No conditional cash transfer programs have been evaluated for their effect on TB outcomes [24,26]. Although several randomized controlled trials have tested the effects of economic incentives in the context of TB ( [25], only one has focused on patients with active TB and only one (the same trial) was conducted in a low-income country [41]. In that study, food supplements to patients on TB treatment were found to have no effect on cure rates. Other non-randomized studies investigating the use of financial incentives in patients with active TB have had varying results. One such project in China, where both patients and providers received cash incentives, showed no impact on TB outcomes [42]. A second project in Cambodia, where patients with TB received nutritional supplementation and participated in a microfinance program, showed improved cure rates in the intervention group [43]. To our knowledge, no studies have tested the impact of economic support on TB outcomes in Africa.

Social and economic interventions to strengthen TB control are rare [12]. In South Africa, patients with TB may be given food parcels when they collect their treatment, and may also be eligible to receive a disability grant. Disability grants, which are income replacement grants, may be given to patients with TB if authorized by a doctor. However, the determination of eligibility for these grants is neither clear nor standardized and varies both between and within provinces [44]. Neither the food parcels nor the disability grants are conditional on any outcomes or behaviors on the part of the patients. Although data on receipt of food parcels and disability grants are not recorded in the TB registers at South African clinics, and were therefore not collected in this trial, we expect that, due to randomization, the proportions of patients receiving them would be the same across intervention and control clinics.

In our study, the lack of a statistically significant effect in the intention to treat analysis may be due in part to the low fidelity to the intervention. It is likely that eligible patients who did not receive any vouchers at all were considered by nurses not to need them. Nurses in the trial, who are used to rationing food supplements to those patients whom they consider most needy, tended to give vouchers out in the same way (process evaluation, to be reported elsewhere). This is illustrated by the finding that unemployed patients in intervention clinics were more likely to receive vouchers than patients who were employed (Table 7). Interestingly, eligible children younger than 13 years were less likely to receive vouchers. Although this seems surprising, it must be noted that the majority of these children would have been in receipt of a child support grant. One of the criteria reported by nurses for not giving eligible patients vouchers was their receipt of other forms of state grants (process evaluation, to be reported elsewhere). Women, who in South Africa are more likely to be poorer than men, were also more likely to receive vouchers.

The analysis of treatment success rates in unemployed patients shows that, within the intervention group, unemployed patients who did receive a voucher achieved better treatment success rates than those who did not. However, reverse causality may be responsible for these findings, as those who received vouchers may have been those who attended the clinics more regularly. Thus this finding should be interpreted with caution, and be investigated in further research.

Further issues that may have contributed to the low fidelity of our trial were the preference of some nurses to give vouchers out in batches at month end, and the logistical difficulties in ensuring that clinics did not run out of vouchers (process evaluation, to be reported elsewhere). That there was no quantification of the impact of these issues on the fidelity to the trial protocol is an important limitation of this trial. More rigorous monitoring of our intervention may have improved fidelity, and made it easier for a trial of this size to detect a significant effect. However, an important aim of this pragmatic trial was to assess the feasibility of administering such vouchers under normal public sector clinic conditions [31].

The exploratory analysis, which investigated the effect of the vouchers in eligible patients who received at least one, attempted to ‘estimate maximum achievable treatment effect’ [33] in a particular subgroup of patients. The patients in intervention clinics who received a voucher at least once were systematically different from the patients in intervention clinics who received no vouchers, and so not only is the potential bias in this analysis acknowledged, it can also to a certain extent be described [31]. The patients who received the vouchers were more likely to be unemployed, and therefore more deprived, than those who did not, because of the nurses’ sense that they should give vouchers preferentially to patients who needed them more (process evaluation, to be reported elsewhere). This exploratory analysis suggests that, in patients who received vouchers, they did have a significant effect on treatment outcomes. Although the intention to treat analysis is presented as the main and most important finding of this trial, the exploratory analysis is included because it adds possible explanatory detail to the trial, and because it raises questions for further research. Such research questions include:

•Would it be feasible to deliver these vouchers (or a similar form of economic support) to poorer patients only?

•How feasible would the means testing inherent in such delivery be?

•Would such means testing be susceptible to manipulation and corruption?

•How would other patients react to the targeting of poorer patients for the receipt of a voucher?

•Would the effect demonstrated in the exploratory analysis be replicated or increased if this voucher were only given to more deprived patients?

The findings of the dose–response analysis support those of the exploratory analysis by suggesting that these vouchers have the potential to improve outcomes on TB treatment. The fact that the subgroups of patients who received the vouchers more frequently achieved significantly better TB outcomes than those who received it less frequently implies that higher fidelity to the intervention may produce a significant benefit. In addition, the dose–response analysis argues against a perverse incentive effect of the voucher. If patients did try to remain ill in order to continue receiving the voucher, treatment success rates would have fallen with frequency and duration of receipt. This is an important finding, given the local and global concern about this unintended consequence of conditional cash transfers, economic incentives and results-based financing [23]. However, reverse causality cannot be ruled out here: it is possible that patients who came to the clinic more often of their own accord were more likely to receive vouchers, and that it was their own motivation to adhere that was responsible for their improved outcomes on treatment, rather than the vouchers *per se*. Future studies should investigate this phenomenon further.

Subgroup analysis showed that treatment success rates were better in women, patients who were employed, children under 13 years of age, and smear-positive patients. Although such results are not found in all settings, they do reflect the findings of many other studies in Africa and elsewhere. In the African [45] and South African contexts [46], women have been shown to have better adherence to TB treatment. However, women in some settings may need to seek permission to attend clinics and may therefore have poorer adherence than men [14]. In our study setting, employed patients appeared to be better off financially than those who were unemployed (assessment of patient poverty, to be reported elsewhere). Although unemployed patients were more likely to receive the voucher than those who were employed, it is likely that the value of the voucher was too small to overcome the barriers to adherence imposed by unemployment and consequent poverty (process evaluation, to be reported elsewhere). The poorer outcomes of unemployed patients are reflected in the findings of several studies in Africa and elsewhere, where low income has been shown to be associated with poorer adherence to TB treatment [14,45]. Further trials should investigate the effect of greater values of economic support on TB treatment outcomes, as well as possible confounders that might affect this relationship. In our study, children younger than 13 years had better outcomes on treatment than those older than 13. Although in some contexts adherence of children to TB treatment is low [47,48], adherence of children in South Africa has been shown to be high [49]. Finally, smear-positive TB was associated in our study with better outcomes on treatment. A possible explanation for this is that these patients are less likely to be infected with HIV, which has been identified as an independent risk factor for default from TB treatment [45,46].

The omission of HIV from the analysis of patients’ responses to the vouchers, due to the lack of data on participating patients’ HIV status and treatment, is an important limitation of this trial. The co-infection rate of TB and HIV in KwaZulu-Natal is high and both the incidence and the geographical distribution of TB in the country have been affected profoundly by HIV. Importantly, under-nutrition, TB and HIV also seem to act synergistically, thus creating the ‘perfect storm’ for epidemics in South Africa [50]. The high co-infection rate of HIV and TB, and the effect of poverty on adherence to treatment for both diseases, make it possible that patients infected by HIV in this trial may have benefited even more from these vouchers than those uninfected by the virus. It is imperative that future research in this field investigate whether and how co-infection with HIV modifies the impact of economic support for patients on TB treatment.

## Conclusions

Our pragmatic trial has shown that, in the real world setting of public sector clinics in South Africa, economic support to patients with TB does not significantly improve outcomes on treatment. Our results suggest that factors related to the administration of such support may undermine its effectiveness. The low fidelity to the delivery of this voucher meant that only a third of all eligible patients received it for four months or more. However, among patients in intervention clinics who received the voucher at least once, treatment success rates were significantly improved. Further, the more frequently the vouchers were received by patients, the higher their probability of treatment success. Further operational research is needed to explore how best to ensure the consistent and appropriate delivery of such support to those eligible to receive it, and whether, under conditions of higher fidelity, the extent of the benefit on treatment outcomes found in this study can be increased.

## Abbreviations

ELISA: Enzyme-linked immunosorbent assay; HIV: Human immunodeficiency virus; GLM: Generalized linear model; PCR: Polymerase chain reaction; TB: Tuberculosis.

## Competing interests

The authors declare that they have no competing interests.

## Authors’ contributions

EL conceptualized the trial, designed the data collection tools, monitored data collection for the whole trial, wrote the statistical analysis plan, cleaned the data, and drafted and revised the paper. SL and JV conceptualized the trial, designed the data collection tools, wrote the statistical analysis plan, and drafted and revised the paper. IF conceptualized the trial and drafted and revised the paper. CL wrote the statistical analysis plan, cleaned the data, analyzed the data and drafted and revised the paper. All authors read and approved the final manuscript.

## Supplementary Material

Additional file 1 Description of pragmatic/explanatory approach to this trial. Click here for file

Additional file 2 Outcome definitions. Click here for file
