# Supplementary material for: Economic support to improve tuberculosis treatment outcomes in South Africa: a pragmatic cluster-randomized controlled trial
Source: Trials. 2013 May 28;14:154. doi: 10.1186/1745-6215-14-154 (PMC3680200; doi:10.1186/1745-6215-14-154)
Supplement: Additional file 1 — Description of pragmatic/explanatory approach to this trial. [file 1745-6215-14-154-S1.doc]

**Additional file 1**

Description of pragmatic/explanatory approach to this trial

| **Domain** | **Predominant approach** | **Description** |
| --- | --- | --- |
| Participant eligibility  criteria | Pragmatic | ‘All participants who have the condition of interest are enrolled, regardless of their anticipated risk, responsiveness, comorbidities or past compliance’. |
| Experimental  intervention —  flexibility | Pragmatic-explanatory | Instructions given for each element of voucher delivery, but practitioners were not rigorously monitored in this. |
| Experimental  intervention —  practitioner  expertise | Pragmatic | ‘The experimental intervention typically is applied by the  full range of practitioners and in the full range of  clinical settings, regardless of their expertise, with only  ordinary attention to dose setting and side effects’. |
| Comparison  intervention —  flexibility | Pragmatic | Routine TB care |
| Comparison  intervention —  practitioner  expertise | Pragmatic | ‘The comparison intervention typically is applied by the  full range of practitioners and in the full range of  clinical settings, regardless of their expertise, with only  ordinary attention to their training, experience and  performance.’ |
| Follow-up intensity | Pragmatic | ‘No formal follow-up visits of study individuals. Instead,  administrative databases … are  searched for the detection of outcomes’. |
| Primary trial  outcome | Pragmatic | ‘The primary outcome is an objectively measured, clinically meaningful outcome to the study participants. The outcome does not rely on central adjudication and  is one that can be assessed under usual conditions (e.g., special tests or training are not required)’. |
| Participant  compliance with  ‘prescribed’  intervention | Explanatory | Patient compliance is measured and is used as criterion for further receipt of vouchers. |
| Practitioner  adherence to study  protocol | Pragmatic-explanatory | Practitioners are encouraged to adhere to study protocol but meetings only take place every 4 to 6 weeks and there is no censure for failing to adhere. |
| Analysis of primary  outcome | Explanatory | ‘An intention-to-treat analysis is usually performed. However, this may be supplemented by a per-protocol  analysis or an analysis restricted to ‘compliers’ or other subgroups in order to estimate maximum achievable treatment effect’. |

Adapted from Thorpe KE, Zwarenstein M, Oxman AD, Treweek S, Furburg CD, Altman DG *et al*. A pragmatic-explanatory continuum indicator summary (PRECIS): a tool to help trial designers. *Journal of Clinical Epidemiology* 2009, **62**:464-475.
