# Supplementary material for: Economic support to improve tuberculosis treatment outcomes in South Africa: a pragmatic cluster-randomized controlled trial
Source: Trials. 2013 May 28;14:154. doi: 10.1186/1745-6215-14-154 (PMC3680200; doi:10.1186/1745-6215-14-154)
Supplement: Additional file 2 — Outcome definitions. [file 1745-6215-14-154-S2.docx]

**Additional file 2: Outcome definitions**

- **TREATMENT SUCCESS: ‘**A patient who was cured or who completed treatment’ (WHO 2009)
- **CURED: ‘**A patient who was initially smear-positive and who was smear negative in the last month of treatment and on at least one previous occasion’ (WHO 2009)
- **COMPLETED TREATMENT: ‘**A patient who completed treatment but did not meet the criteria for cure or failure. This definition applies to pulmonary smear-positive and smear-negative patients and to patients with extra-pulmonary disease (WHO 2009)
- ‘**TREATMENT INTERRUPTION:** ‘Cessation of treatment for less than two months’ (WHO 2007)
- **TREATMENT FAILURE: ‘**A patient who was initially smear-positive and who remained smear-positive at month 5 or later during treatment’ (WHO 2009)
- **DEFAULTED: ‘**A patient whose treatment was interrupted for 2 consecutive months or more’ (WHO 2009)
- **DIED: ‘**A patient who died from any cause during treatment’ (WHO 2009)
- **TRANSFERRED OUT: ‘**A patient who transferred to another reporting unit and for whom the treatment outcome is not known’ (WHO 2009)
- **MDR TB:** Infection with bacilli resistant to at least rifampicin and isonizid treatment (WHO 2007b).

**References**

World Health Organisation. Tuberculosis care with TB-HIV co-management : Integrated Management of Adolescent and Adult Illness (IMAI).Who Geneva, 2007. Available at [http://www.who.int/hiv/pub/imai/TB_HIVModule23.05.07.pdf. Accessed on 27/01/2012](http://www.who.int/hiv/pub/imai/TB_HIVModule23.05.07.pdf.%20accessed%20on%2027/01/2012).

World Health Organisation(b). The Global MDR-TB & XDR-TB Response Plan 2007–2008. WHO Geneva, 2007. Available at http://whqlibdoc.who.int/hq/2007/who_htm_tb_2007.387_eng.pdf

World Health Organisation. Global tuberculosis control: epidemiology, strategy, financing. WHO Geneva, 2009.
